# 4-Methyl­anilinium nitrate

**DOI:** 10.1107/S1600536810005441

**Published:** 2010-03-13

**Authors:** Rui-jun Xu

**Affiliations:** a Ordered Matter Science Research Center, College of Chemistry and Chemical Engineering, Southeast University, Nanjing 210096, People’s Republic of China

## Abstract

In the crystal structure of the title compound, C_7_H_10_N^+^·NO_3_^−^, N—H⋯O hydrogen bonds involving the ammonium group and the nitrate O atoms result in the formation of zigzag chains propagating in [100].

## Related literature

For dielectric-ferroelectric materials, including organic ligands and metal-organic coordination compounds, see: Hang *et al.* (2009 ▶); Li *et al.* (2008 ▶). 
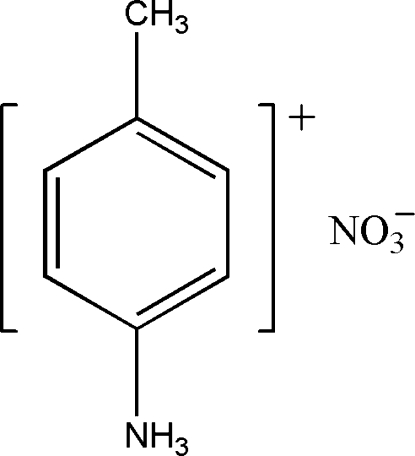


## Experimental

### 

#### Crystal data

C_7_H_10_N^+^·NO_3_^−^
*M* *_r_* = 170.17
Monoclinic, 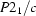

*a* = 5.6468 (11) Å
*b* = 8.7860 (18) Å
*c* = 17.811 (4) Å
β = 99.01 (3)°
*V* = 872.8 (3) Å^3^
*Z* = 4
Mo *K*α radiation
μ = 0.10 mm^−1^
*T* = 298 K
0.60 × 0.40 × 0.40 mm

#### Data collection

Rigaku Mercury2 diffractometer
Absorption correction: multi-scan (*CrystalClear*; Rigaku, 2005 ▶) *T*_min_ = 0.5, *T*_max_ = 0.5
8641 measured reflections
2011 independent reflections
1432 reflections with *I* > 2σ(*I*)
*R*_int_ = 0.038

#### Refinement

*R*[*F* ^2^ > 2σ(*F* ^2^)] = 0.057
*wR*(*F* ^2^) = 0.156
*S* = 1.01
2011 reflections
111 parameters
H-atom parameters constrained
Δρ_max_ = 0.24 e Å^−3^
Δρ_min_ = −0.26 e Å^−3^

### 

Data collection: *CrystalClear* (Rigaku, 2005 ▶); cell refinement: *CrystalClear* data reduction: *CrystalClear*; program(s) used to solve structure: *SHELXS97* (Sheldrick, 2008 ▶); program(s) used to refine structure: *SHELXL97* (Sheldrick, 2008 ▶); molecular graphics: *SHELXTL* (Sheldrick, 2008 ▶); software used to prepare material for publication: *PRPKAPPA* (Ferguson, 1999 ▶).

## Supplementary Material

Crystal structure: contains datablocks I, global. DOI: 10.1107/S1600536810005441/su2140sup1.cif

Structure factors: contains datablocks I. DOI: 10.1107/S1600536810005441/su2140Isup2.hkl

Additional supplementary materials:  crystallographic information; 3D view; checkCIF report

## Figures and Tables

**Table 1:** Hydrogen-bond geometry (Å, °)

*D*—H⋯*A*	*D*—H	H⋯*A*	*D*⋯*A*	*D*—H⋯*A*
N1—H1*A*⋯O1^i^	0.89	2.38	3.138 (3)	143
N1—H1*A*⋯O2^i^	0.89	2.13	2.975 (2)	158
N1—H1*B*⋯O1^ii^	0.89	1.97	2.848 (2)	171
N1—H1*C*⋯O2^iii^	0.89	1.95	2.825 (2)	169

Symmetry codes: (i) 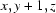; (ii) 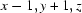; (iii) 
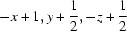
.

**Fig. 1.**
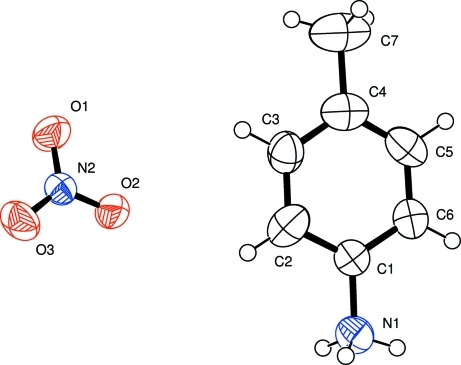
The molecular structure of the title compound, showing the atomic numbering scheme. Displacement ellipsoids are drawn at the 30% probability level.

**Fig. 2.**
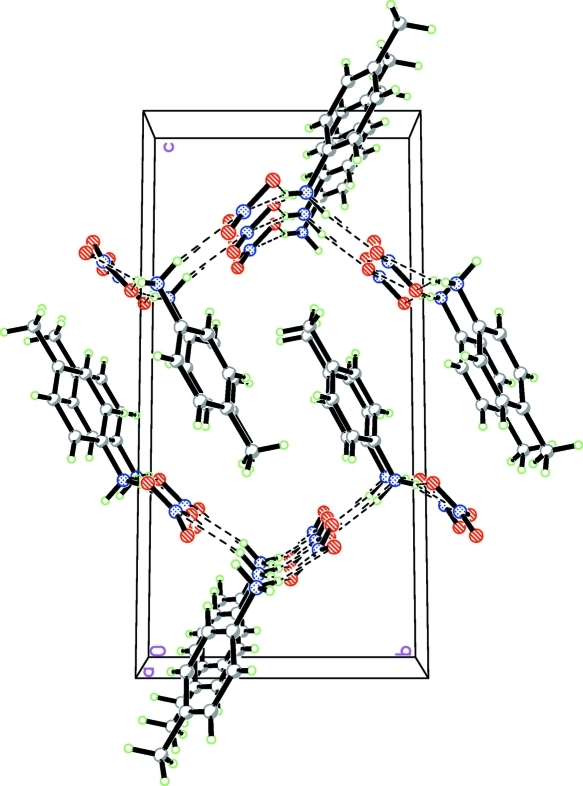
A view of the crystal packing of the title compound. The N—H···O hydrogen bonds are shown as dashed lines (see Table 1 for details).

## supplementary crystallographic information

### Comment

As a part of systematic investigation of dielectric-ferroelectric materials, including organic ligands (Li *et al.*, 2008), metal–organic coordination compounds (Hang *et al.*, 2009), we have found that 4-methylbenzenaminium nitrate has no dielectric disuniform from 80 K to 445 K, (m.p. 465–468 K). Herein we descibe the crystal structure of this compound.

The asymmetric unit of the title compound consists of a 4-methylbenzenaminium cation and a nitrate anion (Fig. 1).

In the crystal N—H···O hydrogen bonds (Table 1) link the cations and anions to form chains propagating along the *a* axis (Fig 2).

### Experimental

The title compound was obtained by mixing p-toluidine and nitric acid in ethanol, in the stoichiometric ratio 1:1. After a few weeks, colorless crystals were obtained by slow evaporation.

### Refinement

The H atoms were included in calculated postions and treated as riding atoms: N—H = 0.89 Å, aromatic C—H = 0.93 Å, methyl C—H = 0.96 Å, with U_iso_(H) = k × U_eq_(parent N- or C-atom), where k = 1.2 for aromatic H atoms, and 1.5 for amonium and methyl H atoms.

### Crystal data

**Table:**

C_7_H_10_N^+^·NO_3_^−^	*F*(000) = 360
*M**_r_* = 170.17	*D*_x_ = 1.295 Mg m^−^^3^
Monoclinic, *P*2_1_/*c*	Mo *K*α radiation, λ = 0.71073 Å
Hall symbol: -P 2ybc	Cell parameters from 3553 reflections
*a* = 5.6468 (11) Å	θ = 3.3–27.6°
*b* = 8.7860 (18) Å	µ = 0.10 mm^−^^1^
*c* = 17.811 (4) Å	*T* = 298 K
β = 99.01 (3)°	Prism, colourless
*V* = 872.8 (3) Å^3^	0.60 × 0.40 × 0.40 mm
*Z* = 4	

### Data collection

**Table:**

Rigaku Mercury2 diffractometer	2011 independent reflections
Radiation source: fine-focus sealed tube	1432 reflections with *I* > 2σ(*I*)
graphite	*R*_int_ = 0.038
Detector resolution: 13.6612 pixels mm^-1^	θ_max_ = 27.5°, θ_min_ = 3.3°
CCD profile fitting scans	*h* = −7→7
Absorption correction: multi-scan (*CrystalClear*; Rigaku, 2005)	*k* = −11→11
*T*_min_ = 0.5, *T*_max_ = 0.5	*l* = −23→23
8641 measured reflections	

### Refinement

**Table:**

Refinement on *F*^2^	Primary atom site location: structure-invariant direct methods
Least-squares matrix: full	Secondary atom site location: difference Fourier map
*R*[*F*^2^ > 2σ(*F*^2^)] = 0.057	Hydrogen site location: inferred from neighbouring sites
*wR*(*F*^2^) = 0.156	H-atom parameters constrained
*S* = 1.01	*w* = 1/[σ^2^(*F*_o_^2^) + (0.0752*P*)^2^ + 0.2152*P*] where *P* = (*F*_o_^2^ + 2*F*_c_^2^)/3
2011 reflections	(Δ/σ)_max_ < 0.001
111 parameters	Δρ_max_ = 0.24 e Å^−^^3^
0 restraints	Δρ_min_ = −0.26 e Å^−^^3^

### Special details

**Table:**

Geometry. Bond distances, angles etc. have been calculated using the rounded fractional coordinates. All su's are estimated from the variances of the (full) variance-covariance matrix. The cell esds are taken into account in the estimation of distances, angles and torsion angles
Refinement. Refinement of *F*^2^ against ALL reflections. The weighted *R*-factor *wR* and goodness of fit *S* are based on *F*^2^, conventional *R*-factors *R* are based on *F*, with *F* set to zero for negative *F*^2^. The threshold expression of *F*^2^ > σ(*F*^2^) is used only for calculating *R*-factors(gt) *etc*. and is not relevant to the choice of reflections for refinement. *R*-factors based on *F*^2^ are statistically about twice as large as those based on *F*, and *R*- factors based on ALL data will be even larger.

### Fractional atomic coordinates and isotropic or equivalent isotropic displacement parameters (Å^2^)

**Table:**

	*x*	*y*	*z*	*U*_iso_*/*U*_eq_	
N1	0.3877 (3)	0.91687 (17)	0.33271 (9)	0.0512 (5)	
C1	0.4679 (3)	0.83868 (18)	0.40462 (10)	0.0434 (5)	
C2	0.6763 (3)	0.7554 (2)	0.41229 (12)	0.0658 (8)	
C3	0.7515 (4)	0.6808 (2)	0.47979 (14)	0.0578 (7)	
C4	0.6214 (4)	0.6858 (2)	0.53968 (12)	0.0607 (7)	
C5	0.4132 (4)	0.7703 (2)	0.52945 (11)	0.0627 (7)	
C6	0.3354 (3)	0.8472 (2)	0.46280 (11)	0.0542 (6)	
C7	0.7061 (5)	0.6011 (3)	0.61266 (14)	0.0920 (10)	
O1	0.9140 (3)	0.02956 (19)	0.33081 (10)	0.0803 (6)	
O2	0.6445 (2)	0.17377 (18)	0.27222 (9)	0.0675 (5)	
O3	0.9986 (3)	0.1724 (2)	0.24110 (9)	0.0737 (6)	
N2	0.8567 (3)	0.12615 (18)	0.28068 (9)	0.0492 (5)	
H1A	0.49500	0.98720	0.32520	0.0770*	
H1B	0.24690	0.96120	0.33430	0.0770*	
H1C	0.37240	0.84980	0.29480	0.0770*	
H2	0.76490	0.74950	0.37260	0.0690*	
H3	0.89360	0.62550	0.48540	0.0790*	
H5	0.32260	0.77550	0.56870	0.0750*	
H6	0.19500	0.90420	0.45730	0.0650*	
H7A	0.57120	0.55570	0.63080	0.1380*	
H7B	0.78420	0.67070	0.65010	0.1380*	
H7C	0.81700	0.52300	0.60350	0.1380*	

### Atomic displacement parameters (Å^2^)

**Table:**

	*U*^11^	*U*^22^	*U*^33^	*U*^12^	*U*^13^	*U*^23^
N1	0.0498 (9)	0.0510 (9)	0.0537 (9)	−0.0069 (7)	0.0109 (7)	0.0010 (7)
C1	0.0431 (9)	0.0401 (9)	0.0470 (9)	−0.0087 (7)	0.0073 (7)	−0.0034 (7)
C2	0.0571 (12)	0.0555 (12)	0.0831 (15)	0.0059 (9)	0.0053 (11)	0.0088 (11)
C3	0.0526 (11)	0.0544 (11)	0.0700 (13)	0.0012 (9)	0.0206 (9)	0.0022 (9)
C4	0.0764 (14)	0.0453 (10)	0.0553 (11)	−0.0150 (10)	−0.0054 (10)	−0.0008 (8)
C5	0.0785 (14)	0.0653 (13)	0.0467 (11)	−0.0118 (11)	0.0175 (10)	−0.0070 (9)
C6	0.0521 (10)	0.0564 (11)	0.0555 (11)	0.0007 (9)	0.0126 (8)	−0.0059 (9)
C7	0.128 (2)	0.0688 (15)	0.0681 (15)	−0.0148 (15)	−0.0194 (15)	0.0106 (12)
O1	0.0720 (10)	0.0797 (10)	0.0930 (12)	0.0197 (8)	0.0244 (9)	0.0394 (9)
O2	0.0512 (8)	0.0811 (10)	0.0718 (10)	0.0102 (7)	0.0143 (7)	0.0174 (8)
O3	0.0618 (9)	0.0937 (12)	0.0703 (10)	−0.0148 (8)	0.0253 (7)	0.0113 (8)
N2	0.0489 (8)	0.0516 (8)	0.0478 (8)	−0.0026 (7)	0.0098 (6)	−0.0004 (7)

### Geometric parameters (Å, °)

**Table:**

O1—N2	1.238 (2)	C4—C5	1.378 (3)
O2—N2	1.256 (2)	C4—C7	1.509 (3)
O3—N2	1.217 (2)	C5—C6	1.377 (3)
N1—C1	1.461 (2)	C2—H2	0.9300
N1—H1A	0.8900	C3—H3	0.9300
N1—H1B	0.8900	C5—H5	0.9300
N1—H1C	0.8900	C6—H6	0.9300
C1—C6	1.372 (3)	C7—H7A	0.9600
C1—C2	1.374 (2)	C7—H7B	0.9600
C2—C3	1.377 (3)	C7—H7C	0.9600
C3—C4	1.387 (3)		
			
H1B—N1—H1C	109.00	C4—C5—C6	121.92 (19)
C1—N1—H1B	109.00	C1—C6—C5	119.10 (17)
C1—N1—H1C	109.00	C3—C2—H2	121.00
C1—N1—H1A	109.00	C1—C2—H2	121.00
H1A—N1—H1C	109.00	C4—C3—H3	119.00
H1A—N1—H1B	109.00	C2—C3—H3	119.00
O1—N2—O2	116.86 (17)	C4—C5—H5	119.00
O2—N2—O3	121.45 (17)	C6—C5—H5	119.00
O1—N2—O3	121.70 (18)	C1—C6—H6	120.00
C2—C1—C6	120.92 (17)	C5—C6—H6	120.00
N1—C1—C6	120.37 (16)	C4—C7—H7C	109.00
N1—C1—C2	118.71 (17)	H7A—C7—H7C	109.00
C1—C2—C3	118.85 (19)	H7B—C7—H7C	109.00
C2—C3—C4	121.9 (2)	H7A—C7—H7B	109.00
C3—C4—C5	117.31 (19)	C4—C7—H7A	109.00
C3—C4—C7	120.8 (2)	C4—C7—H7B	109.00
C5—C4—C7	121.9 (2)		
			
N1—C1—C2—C3	−179.58 (16)	C2—C3—C4—C5	−0.7 (3)
C6—C1—C2—C3	−0.4 (3)	C2—C3—C4—C7	179.1 (2)
N1—C1—C6—C5	178.92 (16)	C3—C4—C5—C6	0.0 (3)
C2—C1—C6—C5	−0.3 (3)	C7—C4—C5—C6	−179.8 (2)
C1—C2—C3—C4	0.9 (3)	C4—C5—C6—C1	0.5 (3)

### Hydrogen-bond geometry (Å, °)

**Table:**

*D*—H···*A*	*D*—H	H···*A*	*D*···*A*	*D*—H···*A*
N1—H1A···O1^i^	0.89	2.38	3.138 (3)	143
N1—H1A···O2^i^	0.89	2.13	2.975 (2)	158
N1—H1B···O1^ii^	0.89	1.97	2.848 (2)	171
N1—H1C···O2^iii^	0.89	1.95	2.825 (2)	169

Symmetry codes: (i) *x*, *y*+1, *z*; (ii) *x*−1, *y*+1, *z*; (iii) −*x*+1, *y*+1/2, −*z*+1/2.
